# Digital subtraction angiography-guided peripheral nerve stimulation via the foramen rotundum for refractory trigeminal postherpetic neuralgia: a case report and literature review

**DOI:** 10.3389/fneur.2024.1353882

**Published:** 2024-02-29

**Authors:** Qingbang Xu, Fangyuan Zhou, Dong Yang

**Affiliations:** ^1^Department of Pain Medicine, Union Hospital, Tongji Medical College, Huazhong University of Science and Technology, Wuhan, China; ^2^Institute of Anesthesia and Critical Care Medicine, Union Hospital, Tongji Medical College, Huazhong University of Science and Technology, Wuhan, China; ^3^Key Laboratory of Anesthesiology and Resuscitation (Huazhong University of Science and Technology), Ministry of Education, Wuhan, China

**Keywords:** foramen rotundum, maxillary division, peripheral nerve stimulation, trigeminal postherpetic neuralgia, case report

## Abstract

Postherpetic neuralgia (PHN) is a debilitating complication of varicella-zoster virus infection. This case report presents a novel approach to treating refractory trigeminal maxillary postherpetic neuralgia using digital subtraction angiography (DSA)-guided peripheral nerve stimulation via the foramen rotundum. A 72-year-old female with severe, treatment-resistant pain underwent this intervention. The results demonstrated the disappearance of tactile allodynia, a significant reduction in oral analgesic requirements, and no observed complications or side effects during a 3-year follow-up period. This case highlights the potential effectiveness of DSA-guided peripheral nerve stimulation using a new dorsal root ganglion (DRG) stimulator as an alternative therapy for refractory trigeminal postherpetic neuralgia (TPHN).

## Introduction

Postherpetic neuralgia (PHN) is a common form of dermatomal pain persisting at least 90 days resulting from herpes zoster (HZ), which is a distinctive syndrome that caused by reactivation of varicella zoster virus (VZV) (1). PHN is conventionally defined as a direct consequence of the response of pathologic damage to nerve tissue from skin to central nervous system during the VZV attack (2, 3). Trigeminal postherpetic neuralgia (TPHN) is a special condition of PHN that is caused by the invasion of VZV in the semilunar ganglion or its branches within the trigeminal nerve. Although it accounts for only 5% of cases of PHN, it remains notoriously resistant to treatment (4–6). Common treatment approaches for TPHN include medication, nerve blocks, destructive procedures, pulsed radiofrequency, and radiofrequency thermoablation (5, 7–10). However, these methods have demonstrated limited effectiveness, particularly in patients with chronic disease.

In past few decades, peripheral nerve electrical stimulation has shown promise in managing trigeminal neuropathic pain resulting from facial trauma or HZ (11). Specifically, pain in the maxillary nerve control area of the trigeminal nerve has been addressed through electrical stimulation of the Gasserian ganglion or infraorbital nerve (12). Unfortunately, these methods present significant drawbacks, including the risk of intracranial hemorrhage, infection, cranial nerve injury, electrode displacement, incomplete control of pain distribution, and unintended stimulation of the V1 or V3 branches. These complications can lead to numbness in the corresponding dermatome, motor weakness, reduced corneal reflex, corneal keratitis, and, in severe cases, permanent vision loss (12–14). In this case report, we introduce a novel approach using DSA-guided peripheral nerve stimulation via the foramen rotundum for the treatment of refractory trigeminal postherpetic maxillary nerve neuralgia.

## Case report

### History and examination

**Figure 1:**
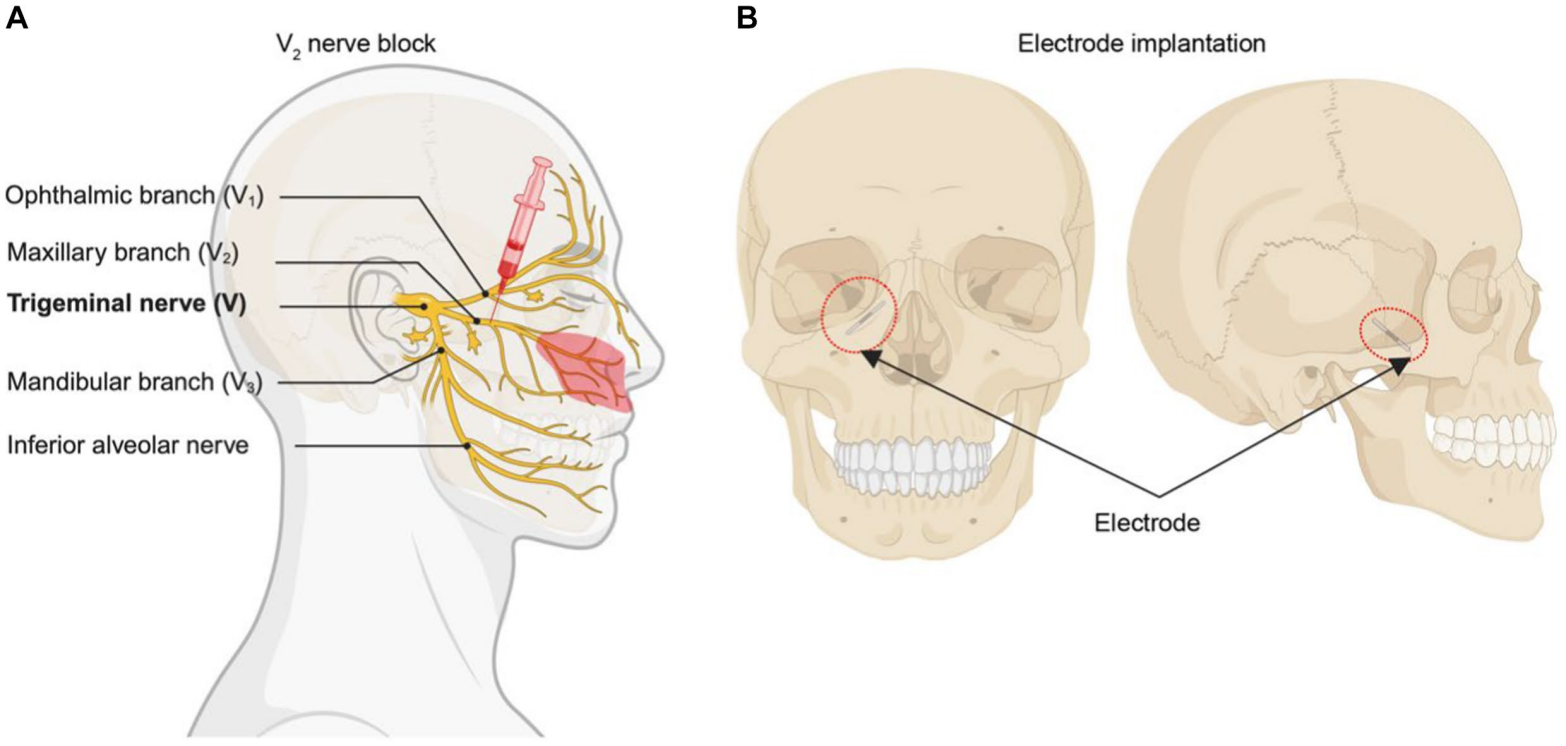
Diagram of surgical procedure. **(A)** Blocking V2 branch of trigeminal nerve with 1% lidocaine. Red region means main allodynia region in the patient. **(B)** The position that the electrical stimulator implant.

This case involves a clinical trial of an implantable spinal cord stimulator system, approved by the Clinical Trial Ethics Committee of Huazhong University of Science and Technology. The patient, a 72-year-old female, had been experiencing prickling sensations in the maxillary area of the trigeminal nerve for 3 years after a right-sided herpes zoster infection. Normal activities such as speaking, brushing teeth, and eating may trigger and exacerbate the pain. The patient did not have any notable medical history of chronic illnesses, immunosuppressive conditions, or psychological disorders. She had a clean record with no prior history of smoking, opioid addiction, alcohol consumption, chronic corticosteroid use, or any other drug dependencies. Physical examination revealed allodynia in the maxillary nerve control area, primarily in the paranasal alar and upper lip (Figure 1A, red region). The patient also exhibited localized superficial hypoesthesia and IV-grade masticatory muscle strength. The Numeric Rating Scale (NRS) score for her pain was 8/10 (0–10). Despite a range of treatments, such as medications including pregabalin (highest dose of 150 mg orally three times daily), oxycodone/paracetamol tablets (highest dose four pills daily), gabapentin (highest dose 1,200 mg daily), and non-steroidal drugs (Celecoxib, highest does 200 mg two times daily), pulsed radiofrequency (a 50 kHz current is delivered in 20 ms pulses at a frequency of 2 Hz for 120 s, in a total of three cycles), and radiofrequency ablation (temperature at 75°C for a duration of 90 s), the pain provided only temporary relief but lacked lasting efficacy. So peripheral nerve field stimulation (PNFS) was initially discussed as a next treatment option.

The success of the nerve block was a prerequisite for electrical stimulation. After obtaining informed consent from the patient, we administered a 1% lidocaine (2 mL in total) maxillary nerve block, which led to effective pain relief (Figure 1A). The surgery is done in two stages, which is similar to the steps in spinal cord stimulation. During the first stage, a trial PNFS-stimulating electrode (Implantable DRG Electrode SCL-302E, Rishena Medical, Changzhou, China) was inserted through the foramen rotundum in the vicinity of the maxillary nerve branch (Figure 1B). Postoperatively, there was a remarkable reduction in the NRS pain score, decreasing from 8/10 to a range of 1–3/10. Tactile allodynia vanished, and there was a significant reduction in the required oral medication dosage. Ten days later, the second stage of the procedure was performed where a permanent electrode was anchored with a permanent electrode (Implantable DRG Electrode SCL-302E, Rishena Medical, Changzhou, China) and tunneled to an implantable pulse generator (Implantable Spinal Cord Stimulator SCS-301A, Rishena Medical, Jiangsu, China).

### Surgical technique procedure

#### Trial stimulation (Stage I)

**Figure 2:**
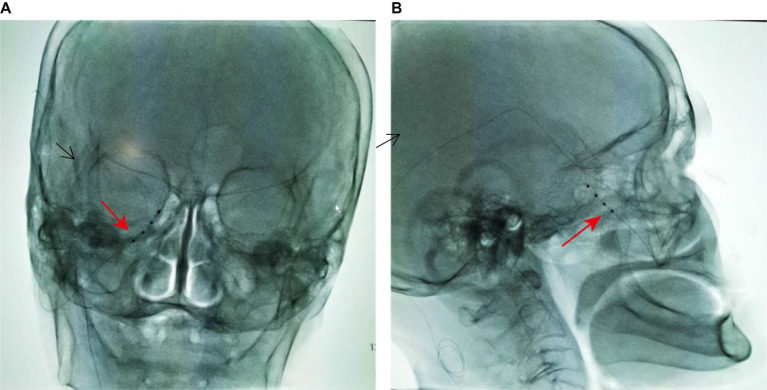
Radiographs of the maxillary branch electrode implanted through the foramen rotundum region (arrow). **(A)** Skull anteroposterior, C-arm angulation (LAO 10°, CRAN 6°). **(B)** Skull lateral, C-arm angulation (LAO 106°, CRAN 3°).

The patient was positioned supine with a thin pillow under the shoulder; the head was rotated with 15° toward the side opposite the painful area. A C-arm was positioned around the patient’s head to allow an anteroposterior view of the head during electrode insertion. Other preparation included placing nasal oxygen catheter, vital signs monitoring, and establishing venous access. After sterile preparation and draping, 1% lidocaine (5 mL) was used for local infiltration anesthesia for insertion. A 14-Ga Tuohy needle was inserted at the point of intersection between a horizontal line at the base of the nose and a vertical line 1 cm from the lateral canthus (15). Radiographic guidance with appropriate angles allowed precise placement of the trial electrode. When inserting the needle, the tip was directed toward the target positioned by DSA. As the needle tip approached the upper part of the pterygopalatine fossa, patient experienced a shock-like pain in the innervation area of the maxillary nerve. Lateral and anteroposterior imaging confirmed that the needle tip was near the foramen rotundum external opening. About 6.5 cm of depth was inserted from skin to foramen rotundum (see Figure 2). After the electrode was placed in the desired location, stimulation was conducted to confirm the correct positioning of the electrode. The electrode was coiled around the entry point. Finally, the electrode was anchored to the skin covered with sterile dressing and connected to the screening device cable. The technician programmed stimulation parameters for delivering electrical stimulation through an external stimulator connected to a percutaneous stimulation lead. The stimulation, set between the first two contacts at 40 Hz with a pulse width of 200 μs, the current intensity between 250 and 350 μA was adjusted according to the sensation of patient. After implantation, the patient was instructed to handle and adjust the stimulation voltage. The trial with more than 50% pain relief was considered successful.

#### Implantation (Stage II)

**Figure 3:**
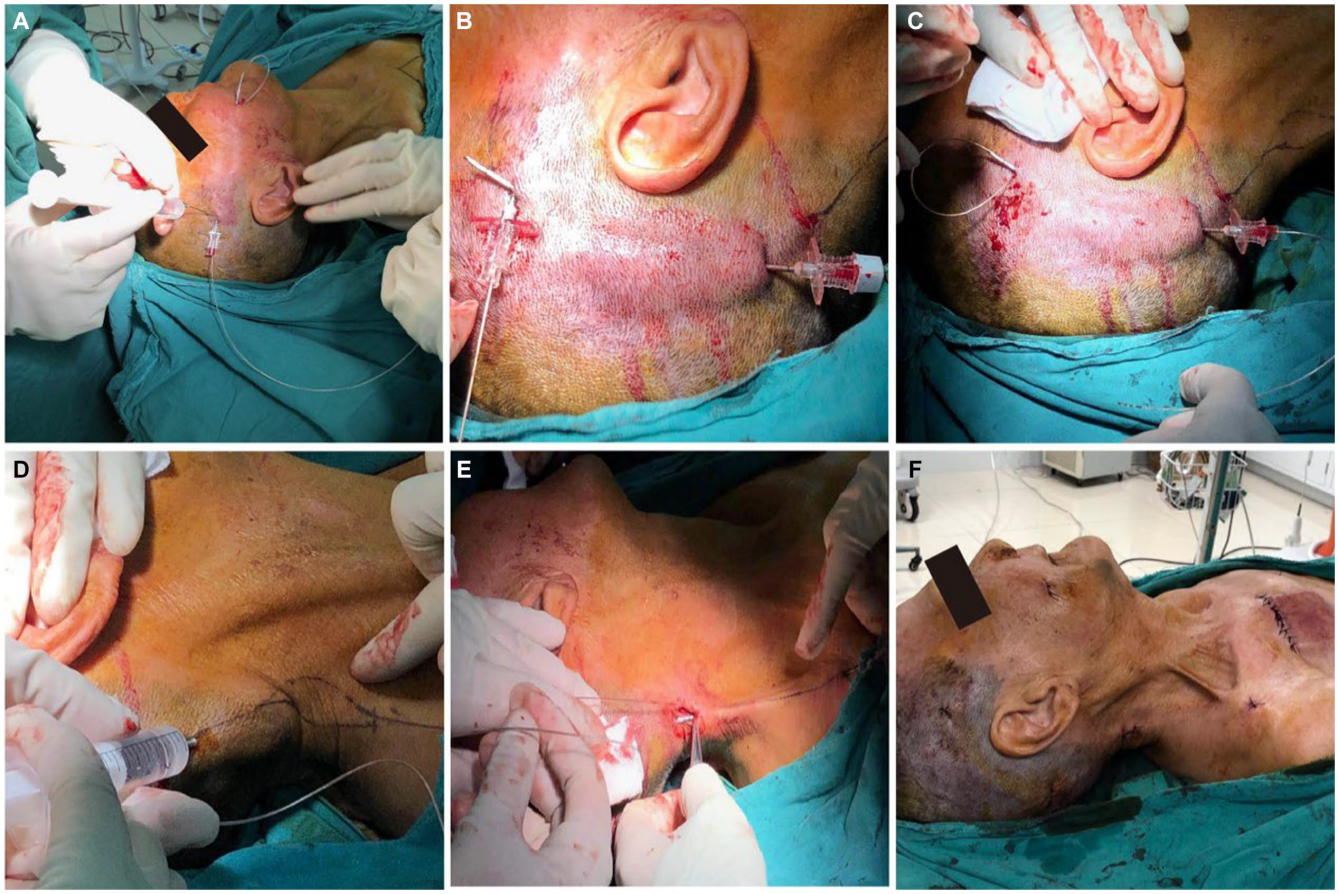
The key point of surgical steps for stage II. **(A–E)** The electrode is then tunneled from the entry point to IPG site using “needle-over-the-stylet” technique. **(F)** The implantable pulse generator was positioned within a subcutaneous pocket beneath the clavicle.

Following the successful trial phase, a permanent stimulation system was implanted 10 days after trial stimulation. The permanent electrode was implanted using a similar procedure to the trial phase, maintaining the same insertion point. A superior auricular incision was carefully created, and the electrode’s rear end was delicately guided toward it utilizing a “needle-over-the-stylet” technique that was described by Slavin and Wess (12). Briefly, the stylet was directed toward the retroauricular incision, followed by the passage of the needle over the stylet. Subsequently, the electrode was threaded into the needle, and the needle was then removed, moving the electrode toward its anchoring point behind the ear. To secure the electrode in place, regular plastic anchors provided in the kit and nonabsorbable sutures were utilized. Extending from the retroauricular incision, the extension cables were tunneled toward a secondary 2 cm incision made in the posterior area of the right neck. Finally, in a manner reminiscent of tunneling extension cables for a spinal cord stimulator, the extension cables were routed toward a 6 cm incision beneath the clavicle. Once all connections were firmly established, the implantable pulse generator was positioned within a subcutaneous pocket beneath the clavicle and anchored to the thoracic fascia with nonabsorbable sutures (Figure 3).

### Follow-up and outcome analysis

**Figure 4:**
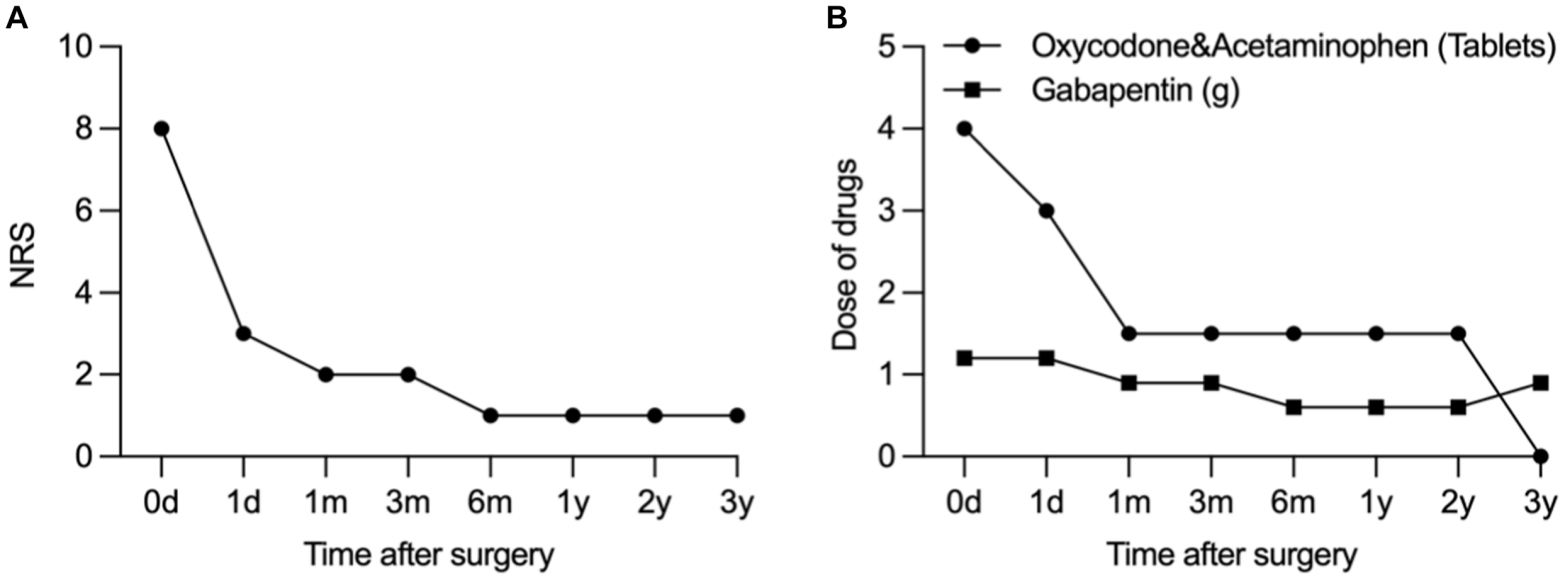
Change in NRS **(A)** and dose of drug **(B)** from the preoperative evaluation to postoperative period at 1-day, 1-month, 3-month, 6-month, and 1–3-year.

Postoperatively, there was a remarkable reduction in the NRS pain score, decreasing from 8/10 to a range of 1–3/10. Tactile allodynia vanished, and there was a significant reduction in the required oral medication dosage. Ten days later, we performed permeant peripheral nerve electrical stimulator implant. Figure 4 illustrates the change in NRS scores and drug dosage from the preoperative evaluation to the 1-day, 1-month, 3-month, and 6-month postoperative periods. The results demonstrate significant pain reduction and reduced reliance on oral medications for pain management.

## Discussion

Refractory trigeminal postherpetic neuralgia presents a formidable challenge in the realm of orofacial neuropathic pain, characterized by recurrent, unilateral, and intense paroxysmal pain episodes (16). Back in 1967, peripheral nerve stimulation emerged as a potential treatment for intractable trigeminal neuralgia (17). Nevertheless, the complexity, likely involving a sophisticated interplay between the central and peripheral nervous systems (11, 18, 19). Peripheral nerve electrical stimulation was traditionally applied near the infraorbital or supraorbital nerve for trigeminal nerve control. Unfortunately, this approach was marred by challenges such as electrode displacement, infections, local wound healing complications, and incomplete pain relief, thus limiting its widespread use (18–20). An alternative approach, the percutaneous trigeminal gessererian ganglion stimulation, has long been a classical method for treating primary trigeminal nerve pain. However, it comes fraught with the risks of injury, intracranial hemorrhage, and infection (6, 10, 21–23). Recent studies have demonstrated that utilizing DSA or CT-guided punctures can provide direct access to the foramen rotundum, reducing the risk of skull penetration and infection when treating primary trigeminal nerve pain in the maxillary nerve control area (12). The foramen rotundum is a bony canal with a diameter of 3 mm and a length of 3–6 mm, and its long axis runs predominantly from the inside to the outside of the skull, oriented in a posterior, upward, frontal, and downward direction (24). The procedure involved inserting with a 14G DRG electrode needle (2.1 mm diameter and 115 mm length, with a sleeve of 1.3 mm outer diameter and 1.0 mm inner diameter) through the foramen rotundum. A recent case report by Huang et al. placed this kind of DRG stimulator into the lateral epidural space adjacent to dorsal root ganglion at L4 level and achieved satisfactory control of pain and this DRG electrode can be accurately implanted into nerve root ganglia, peripheral nerves, etc. through the narrow spaces such as intervertebral foramen, sacral foramen, foramen rotundum, and foramen ovale (25). Compared to traditional spinal cord electrode, this peripheral nerve electrode is smaller and flexible that less likely compress the maxillary nerve or cause leakage of cerebrospinal fluid when passes through the foramen rotundum. Although limited research has delved into the effects of peripheral nerve electrical stimulation on the trigeminal nerve, it offers a lower risk profile and fewer complications while still achieving therapeutic effects (17, 26, 27).

A search of Medline (1966 to second week October 2023), Excerpta Medica Database (EMBASE) (1980 to present), and the Cochrane Database was performed using the following search terms: trigeminal postherpetic neuralgia, postherpetic neuralgia, neuropathic craniofacial pain, and peripheral nerve stimulation. We found 19 cases of TPHN treated with PNS (Table 1). Treatment of TPHN with PNS have been reported in literature rarely, especially the single V2 branch (Table 1). Most published reports on the use of PNS for TPHN treatment have shown significant improvement in pain intensity (Table 1). Slavin et al. (12) started to use peripheral trigeminal stimulation for treatment of intractable trigeminal neuropathic pain but not for trigeminal neuralgia since 1999. Of note, Feletti et al.(32) pointed that when inserting the electrode, the area of allodynia should not be selected as the target area, because a complete deafferentation usually prevents any effect of PNFS or can even paradoxically enhance pain. Instead, the electrode should be positioned at the hyperalgesia peripheral area, where nerve connections are still present. Another study reported that eight patients who underwent electrical stimulation of the supraorbital nerve and infraorbital nerve achieved NRS scores ranging from 0 to 3 post-operation, with some patients experiencing complete pain relief but still experienced pain in the maxillary nerve control area, suggesting the possibility of incomplete pain relief and common complications associated with peripheral nerve electrical stimulation (36). Postherpetic neuralgia seems to be particularly resistant to treatment with gasserian ganglion stimulation, even though the sensory loss is generally subtotal. Of 17 patients with trigeminal postherpetic neuralgia reported in previous studies, only two obtained significant relief (21). Thus, our study chose to focus on electrical stimulation of the maxillary nerve through the foramen rotundum.

**Table 1:** Literature review of the cases with trigeminal postherpetic neuralgia treated with peripheral nerve stimulation.

Author (year)	Cranial nerve site	Cases	Procedure	Outcome
Dunteman et al. (28)	V1	2	PNS on SON	Greatly improved
Johnson et al. (29)	V1	4	PNS on SON	Pain free
Slavin et al. (12)	V1 or V3	3	PNS on ION or SON	Pain relief >50%
Upadhyay et al. (30)	V1	1	PNS on SON	Excellent
Stidd et al. (31)	V1	1	PNS on SON	Pain relief 60%
Feletti et al. (32)	V1	1	PNS on SON	Greatly improved
Ellis et al. (33)	V1	1		Improved
	V1, V2	2		Improved
	V2, V3	1		Not improved
Lerman et al. (34)	V1	1	PNS on STN/SON	Pain relief >90%
Zhao et al. (35)	V2, V3	1	Cervical SCS/PNS on SON	Pain relief >90%
	V1, V2	1	PNS on STN/SON	Pain relief >95%

STN, Supratrochlear nerve stimulation; SON, Supraorbital nerve; ION, Infraorbital nerve; PNS, Peripheral nerve stimulation; and SCS, Spinal cord stimulation.

Our study results, within 3-year post-operation, indicated a successful reduction of pain in the affected area, with no pain triggered by activities such as speaking, brushing teeth, or eating. Allodynia disappeared, and walking-induced pain became tolerable, exerting minimal impact on daily life. Over time, the oral medication dosage gradually decreased, and no complications, such as electrode migrations or infections, were observed. These findings underscore the therapeutic effectiveness and safety of peripheral nerve electrical stimulation through the foramen rotundum. However, it is important to acknowledge the limitations of this study, including the need for longer-term observations and only reporting of a single case. Future research endeavors should encompass extended follow-up to confirm the durability of these benefits, and well-designed, large-scale clinical trials are warranted to comprehensively assess the safety and effectiveness of DSA-guided peri-foramen electrical stimulation implantation for treating refractory herpes-induced trigeminal nerve maxillary branch pain.

## Conclusion

Electrical stimulation of the peripheral nerve through the foramen rotundum using a new DRG stimulator shows satisfying results for trigeminal maxillary branch postherpetic neuralgia. It offers a novel approach for patients with refractory TPHN, which does not respond effectively to traditional therapies.

## Data availability statement

The raw data supporting the conclusions of this article will be made available by the authors, without undue reservation.

## Ethics statement

Written informed consent was obtained from the individual(s) for the publication of any potentially identifiable images or data included in this article.

## Author contributions

QX: Conceptualization, Data curation, Investigation, Methodology, Writing – original draft, Writing – review & editing. FZ: Conceptualization, Data curation, Investigation, Methodology, Software, Writing – original draft, Writing – review & editing. DY: Project administration, Supervision, Validation, Writing – review & editing.
